# Investigation of Porous Metal-Based 3D-Printed Anode GDLs for Tubular High Temperature Proton Exchange Membrane Fuel Cells

**DOI:** 10.3390/ma13092096

**Published:** 2020-05-01

**Authors:** María Catalina Bermúdez Agudelo, Manfred Hampe, Thorsten Reiber, Eberhard Abele

**Affiliations:** 1 Thermal Process Engineering Group (TVT), Institute for Nano- and Microfluidics (NMF), Technical University of Darmstadt, Otto-Berndt-Straße 2, D-64287 Darmstadt, Germany; hampe@tvt.tu-darmstadt.de; 2 Institute of Production Management, Technology and Machine Tools (PTW), Technical University of Darmstadt, Otto-Berndt-Straße 2, D-64287 Darmstadt, Germany; T.Reiber@ptw.tu-darmstadt.de (T.R.); E.Abele@ptw.tu-darmstadt.de (E.A.)

**Keywords:** additive manufacturing, gas diffusion layer (GDL), high-temperature proton exchange membrane fuel cell (HT-PEMFC), MEA preparation, porosity, powder bed fusion using a laser beam (PBF-LB), tubular design

## Abstract

A high-temperature proton exchange membrane fuel cell (HT-PEMFC) conventionally uses a planar design with carbon-based substrates as the gas diffusion layer (GDL) materials. However, the metal-based substrates allow for alternative designs. In this study, the applicability of porous thin-walled tubular elements made of 316L stainless steel as the anode GDL in a multi-layer tubular HT-PEMFC was investigated. The anode GDLs were fabricated via powder bed fusion using a laser beam (PBF-LB) process with defined porosities (14% and 16%). The morphology of the porous elements was compared using scanning electron microscopy (SEM) micrographs. The influence of the porosity on the fuel cell performance was evaluated through electrochemical characterization and a short-term stability test (45 h) in a commercial test station operated at 160 °C and ambient pressure, using hydrogen as the fuel and air as the oxidant. The results showed that the fuel cell manufactured upon the anode GDL with a porosity of 16% had a higher performance with a peak power density of 329.25 W/m^2^ after 5 h of operation at 125.52 A/m^2^ and a voltage degradation rate of 0.511 mV/h over the stability test period. Moreover, this work indicates that additive manufacturing could be a useful tool for further fuel cell development.

## 1. Introduction

The proton exchange membrane or polymer electrolyte membrane fuel cell (PEMFC) can be classified based on the working temperature into low temperature (LT)-PEMFC (usually below 80 °C) and high temperature (HT)-PEMFC (up to 200 °C). Due to its considerable increase in the working temperature, the HT-PEMFC exhibits certain advantages: enhanced electrochemical kinetics, especially the oxygen reduction reaction (ORR); simplified water management systems; increased tolerance to fuel impurities; improved overall efficiency; and simpler system designs [1,2,3,4]. Nevertheless, there are still some key issues and challenges that have delayed the expansion of this type of fuel cell, for instance, membrane electrode assembly (MEA) degradation [2]. These characteristics have motivated researchers all around the globe to investigate and optimize HT-PEMFC systems. From the last year alone, more than 1500 research articles can be found in scientific databases.

Many of these investigations focus on the research and development of the GDL due to its importance for fuel cell performance. The GDLs are responsible for [5,6]: (i) uniformly transporting the reactant gases into the cell and removing products from the catalyst layers (CLs), (ii) giving physical support for the gas diffusion electrode (GDE), (iii) ensuring electric conduction to and from the catalyst layers (CLs), and (iv) providing corrosion resistance. Therefore, the GDL requires an appropriate porosity and proportion of open pores to ensure gas transportation, mechanical stability, and good thermal and electrical conductivity. Usually, carbon-based substrates (CBS) have been employed; however, the metal-based macroporous substrates (MPS) are receiving increased attention, thanks to their high corrosion resistance, high electrical and thermal conductivity, thinner layer thickness, and in certain cases, adjustable porosity. Metal-based MPS (or single metal-based GDLs) are used in the form of metal foams, metal mesh, wire cloth, porous metallic coating, and micro-machined metal mainly made of titanium, nickel, nickel-chromium alloy, and stainless steel [5,6,7,8,9].

Recently, Abele et al. [10] patented a thin-walled porous metal substrate for operating a fuel cell and its preparation via additive manufacturing. Among additive manufacturing processes, the powder bed fusion using a laser beam (PBF-LB) technique offers the possibility of tailoring thin-walled elements with a defined porosity by adjusting the process parameters, e.g., reduced supplied energy, such that the metal powder is not completely melted within and between the layered structure [10,11,12]. The PBF-LB process is not only a fast and economical route for fabricating fuel cell components, but is also a promising candidate for introducing robust and non-conventional designs for GDLs, principally because this technique can produce all possible geometries. For instance, Lang [13] investigated the application of this technology in tubular HT-PEMFC using EN 1.4542 martensitic stainless steel (US: 17-4 PH). With the developed functional fuel cells, a proof of concept was achieved.

Better corrosion resistance is expected if an austenitic stainless steel, such as EN 1.4404 stainless steel (ASTM 316L) (often referred to as 316L), is used. Its austenitic crystalline structure and the inclusion of alloys, such as molybdenum and nickel, improve the chemical resistance relative to other stainless steel groups. In this respect, Abele et al. [14] reported that 316L material has a significantly lower mass loss than 17-4 PH. Similarly, the investigation of Bermúdez et al. [15] showed that the anode GDL from 316L material had a superior performance in comparison to the one fabricated from 17-4H material. Although the 316L has been extensively investigated for the PBF-LB process in marine applications, biomedical prosthesis, and bipolar plates for fuel cells [16,17,18,19,20,21,22,23,24], only one study has been published, to the authors’ knowledge, regarding GDLs [15].

A few studies in the literature indicate that tubular PEMFCs have better gravimetric and volumetric power densities than conventional prototypes [13,25,26]. Additionally, a tubular geometry could suggest a reduction in manufacturing costs since the end plates and/or bipolar plates might be omitted. Regarding the design of fuel cell stacks, the use of single tubular cells could also favor unexplored configurations with smaller sealing areas and better accessibility to exchange defective single cells.

This study presents an investigation of novel 316L stainless steel GDLs (hereafter referred to as anode GDLs) produced using PBF-LB with different porosities on tubular HT-PEMFC under concentrated phosphoric acid (H_3_PO_4_) conditions. The surface morphology of the fabricated anode GDLs was examined using scanning electron microscopy (SEM) analysis. The multilayer manufactured MEAs with theoretically defined porosity of 14% (denoted as FC1_14%) and 16% (denoted as FC2_16%) were characterized through current-voltage measurements (polarization curve), electrochemical impedance spectroscopy (EIS), and short-term stability tests.

## 2. Experimental

### 2.1. Production of Metallic Porous Tubular Anode GDLs

The thin-walled metallic tubular substrates used in this study were manufactured via the PBF-LB technique using a 3D-printing system (EOSINT M270, EOS GmbH, Krailling, Germany). In the PBF-LB process, the porosity of the specimen was controlled by changing the area energy density ($E_{A}$), which in turn is a function of the process parameters: laser power (P), scan speed ($v_{s}$), and hatch spacing ($h_{s}$), through Equation (1). It was found that the higher the area energy density, the lower the porosity obtained [12,14,27].

(1) $$E_{A}=\frac{P}{v_{s}\times h_{s}}$$

Abele et al. [14,27] investigated the influence of these parameters on the porosity for two stainless steel materials, obtaining a porosity range of 0.99–17.35% for 17-4 PH and 0.11–19% for 316L in a suitable process window. Based on this, the porosities of 14% and 16%, which are close to the upper limit, were selected to evaluate the suitability of this 3D-printing technique in GDL production. 

The porosity produced via the PBF-LB process ($\varepsilon_{th}$) is known to be geometrically undefined and was measured following the simple and accurate Archimedes method by measuring the weight and volume of the sample using Equation (2), where $m_{a}$ is the specimen mass in air, $m_{l}$ is the specimen mass in deionized water, $\rho_{l}$ is deionized water density, and $V_{A}$ is the apparent volume of the specimen [28]:

(2) $$\varepsilon_{th}=1-\frac{m_{a}-m_{l}}{\rho_{l}\times V_{A}}.$$

**Figure 1:**
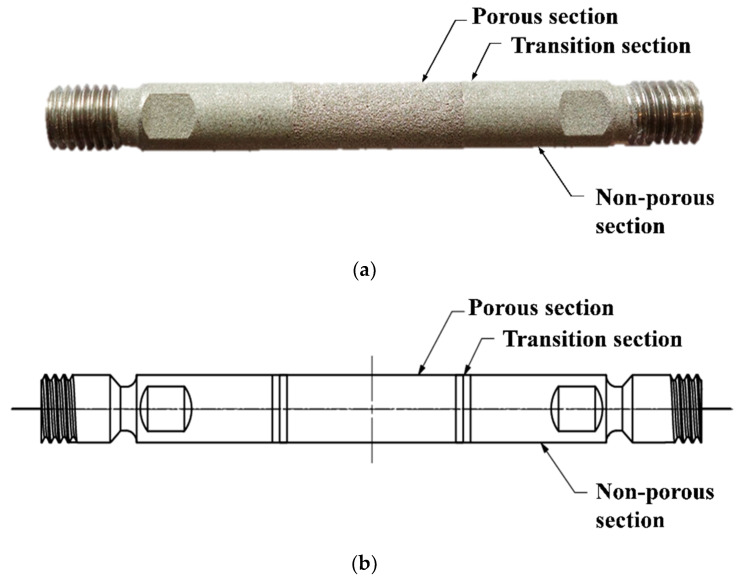
PBF-LB-produced anode electrode: (**a**) prototype and (**b**) section view.

The tubular prototypes, shown in Figure 1, combined a dense segment with a porous structure, which acted as the hydrogen channel, gas diffusion layer, and anode current collector. The porous section corresponded to the active area of the electrode and the non-porous/dense section provided mechanical stability during the assembly. To ensure electrical insulation and gas tightness, the dense section was covered with a heat-shrinking tubing (DERAY KYF 190, DSG-Canusa GmbH, Rheinbach, Germany).

To counteract the surface corrosion and maintain reasonable production costs, the commercial 316L stainless steel material was chosen for further investigation since its austenitic structure, comprising 16.5–18.5 wt % of chromium, 10–13 wt % of nickel, 2–2.5 wt % of molybdenum, and a very low carbon content (<0.03 wt %), has demonstrated good corrosion resistance in harsh chemical environments [29,30,31,32,33,34,35,36,37].

### 2.2. Preparation of the Membrane Electrode Assemblies and Single Tubular Fuel Cell

The multilayer tubular HT-PEMFC was manufactured as follows.

#### 2.2.1. Preparation of Catalyst Layer

Catalyst inks were prepared using a commercial platinum on carbon (Pt-C) catalyst (HiSPEC® 3000, Alfa Aesar, Ward Hill, MA, USA), polytetrafluoroethylene (PTFE) dispersed solution (Dyneon TF 5070 GZ, 3M Company, St. Paul, MN, USA), and isopropyl alcohol/deionized water mixture as a solvent. The powder concentration [Pt-C + PTFE] in each ink was 3 wt % and the mass ratio of Pt-C/PTFE was 50/50 for the hydrogen oxidation reaction (HOR) and 75/25 for the ORR. Before the catalyst coating, the catalyst inks were sonicated for at least 30 min to obtain homogenous solutions. The anode catalyst layer was sprayed onto the PBF-LB-produced porous electrode (GDL-based method) and the cathode layer was sprayed on the membrane (known as catalyst-coated membrane method or CCM method); the platinum loadings were 0.57 ± 0.01 mg/cm^2^ and 0.44 ± 0.03 mg/cm^2^, respectively.

#### 2.2.2. Preparation of the Sol-Gel PBI-H_3_PO_4_ Membrane

The membrane used in this study was prepared using a sol-gel process with a doping level of 35 moles of H_3_PO_4_ per mole polymer repeating unit of polybenzimidazole (PBI). The procedure used for the membrane production was as described by Lang [13]. First, a commercial m-PBI (Celazole® PBI, PBI Performance Products, Inc., Southern Pine Blvd. Charlotte, NC, USA) was mixed with 85 wt % of H_3_PO_4_ at high temperature (>200 °C) to concentrate the acid and generate polyphosphoric acid (PPA) through a polycondensation reaction. Then, the anode GDE (PBF-LB-produced element + anode catalyst layer) was immersed in the PBI/PPA solution for 60 s. Finally, it was lifted out at constant speed. The characteristics of the membranes produced in this way could be influenced by parameters like PPA concentration, dipping temperature, residence time, lifting speed, and relative humidity of the environment. Therefore, all used membranes were coated with the same sol-membrane under identical conditions to guarantee consistency. The sol-gel transition started when the prototype absorbed the moisture from the atmosphere. 

After gelation, the excess acid was removed with deionized water and the prototype was prepared for the cathode catalyst layer application.

#### 2.2.3. Cathode GDL and Electric Connection

The commercial carbon braided hose (KB-4002, Carbon-Werke Weißgerber GmbH & Co KG, Wallerstein, Germany) was used as the outer electrode and the cathode gas diffusion layer. This woven fabric was slid over the active area and fastened with a tungsten wire to provide a homogenous compression throughout the fuel cell and conduct the produced current.

### 2.3. Electrochemical Characterization

Each fabricated single fuel cell, with an active area of 4.78 cm^2^, was placed into a tubular reactor and connected electronically to a commercial fully automated fuel cell station (Evaluator C100, Horiba FuelCon GmbH, Barleben, Germany) for electrochemical characterization. The fuel cell was operated at 160 °C and atmospheric pressure using hydrogen quality 3.0 (I7001L50R2A001, Air Liquide S.A., Paris, France) as the fuel and air as the oxidant, at flow rates of 2.64 cm^3^/s and 52.86 cm^3^/s, respectively.

After a proper conditioning phase, automated quasi-stationary state polarization curves and EIS measurements were conducted. The current-voltage polarization curves were obtained using a combination of both galvanostatic and potentiostatic guide mode with a voltage range from the open-circuit voltage (OCV) to 0.19 V. Each operating point was held for 120 s before recording to guarantee the quasi-stationary state. Individual cell resistances were evaluated through an integrated TrueData-EIS analyzer (impedance accuracy up to 2%, Horiba FuelCon GmbH, BarlebenCity, Germany). The in situ EIS measurements were recorded during fuel cell operation at 0.06 A within a frequency range of 0.1–20,000 Hz in three consecutive sections in a logarithmic apportionment. Additionally, a short-term stability test was conducted at a constant load (0.06 A) for a period of 45 h, interrupted every 5 h, using in situ electrochemical measurements. The degradation rate was calculated based on the voltage reduction along the complete test period.

## 3. Results and Discussion

### 3.1. Surface Morphology of the Produced PBF-LB Test Elements

**Figure 2:**
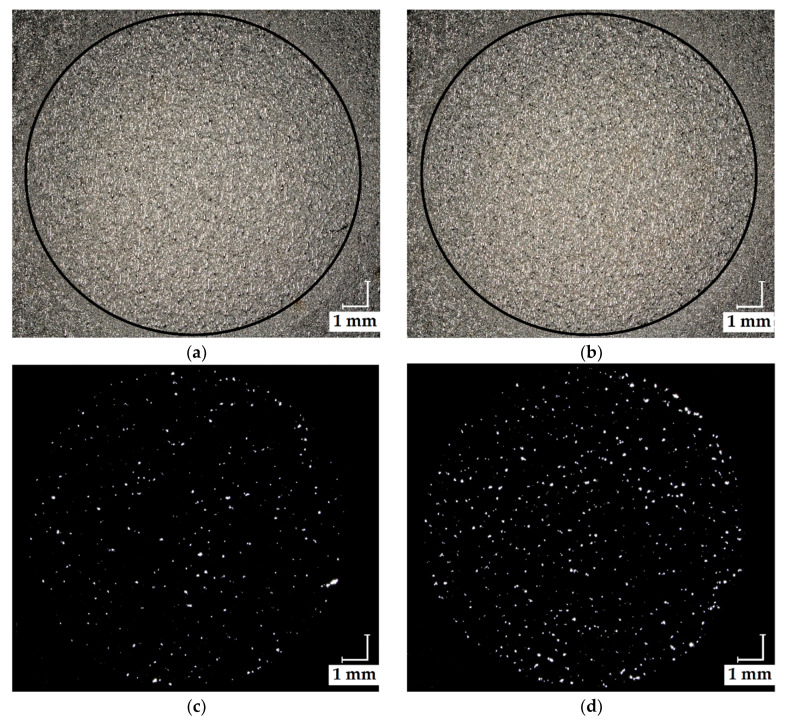
PBF-LB test elements combining dense and porous structures (circle) with porosities: (**a**) 14% and (**b**) 16% with the respective reflected light micrographs (**c**) and (**d**).

Analogous to the active area of the anode GDLs under study, test elements with a wall thickness of 250 μm and defined porosities of 14% and 16% were fabricated, as shown in Figure 2a,b. The test elements were evaluated using reflected light microscopy (Figure 2c,d) and SEM analysis.

**Figure 3:**
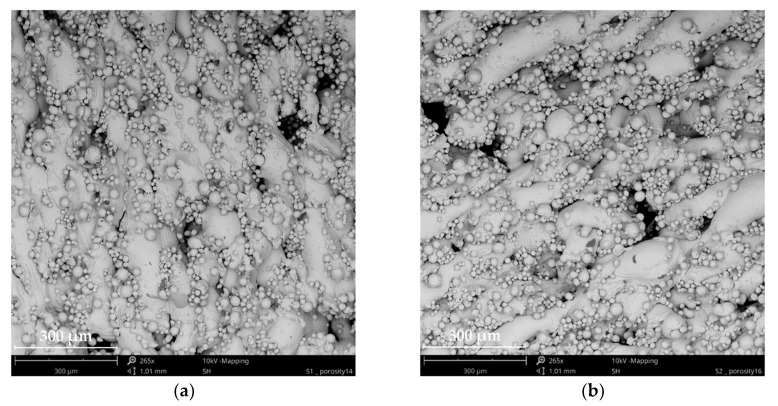
SEM micrographs of the porous structure of the anode GDLs with porosities of (**a**) 14% and (**b**) 16%.

The surface microstructure of the fabricated GDLs was analyzed using SEM images. The top-view micrographs of the samples are presented in Figure 3. The rough surface of the samples provided a high surface area. This increased the possible active places for reactions, but at the same time, it enlarged the tortuosity, slowing down the diffusion of the hydrogen gas through the layer. Since HOR is faster than ORR, this represented a small sacrifice.

The pore type of all evaluated samples exhibited a complex amorphous geometry. The topography images from Figure 3 show that the pore size increased while the distance between the pores decreased with the porosity. Figure 3b displays a higher macro-open-pore structure, which was enough to guarantee a good supply of reactants. However, this arrangement can compromise the mechanical support needed for the CL and the membrane.

**Figure 4:**
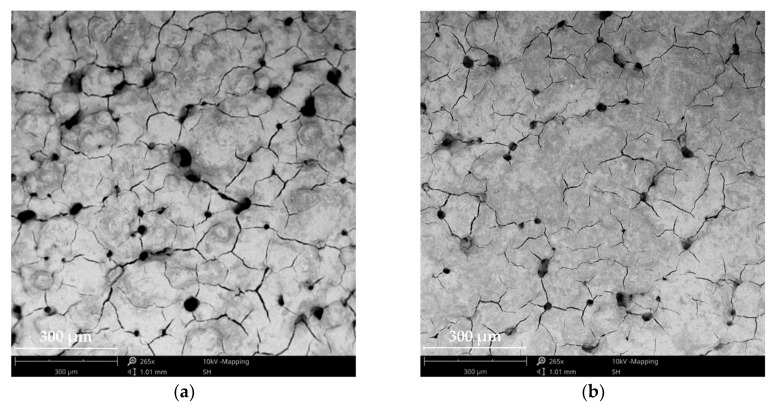
SEM micrographs of the anode GDEs with porosities of (**a**) 14% and (**b**) 16%.

Micrographs of anode GDEs (anode GDL + anode CL) are presented in Figure 4 to qualitatively compare the surface condition of the anode CL relative to the anode GDL. The top view shows a mud crack morphology for both electrodes. The GDEs show opposite behavior to the GDLs, i.e., as the anode GDL porosity increased, the pore size decreased and the distance between pores increased.

As can be seen in Figure 4b, the deposited CL had an even surface with finer agglomerates, whereas the electrode in Figure 4a showed coarse agglomerates. The agglomerates formed on the GDE surface could hinder the gas distribution and decreased the active catalyst area, which in turn led to a significant drop in performance.

### 3.2. Tubular HT-PEMFC Performance

**Figure 5:**
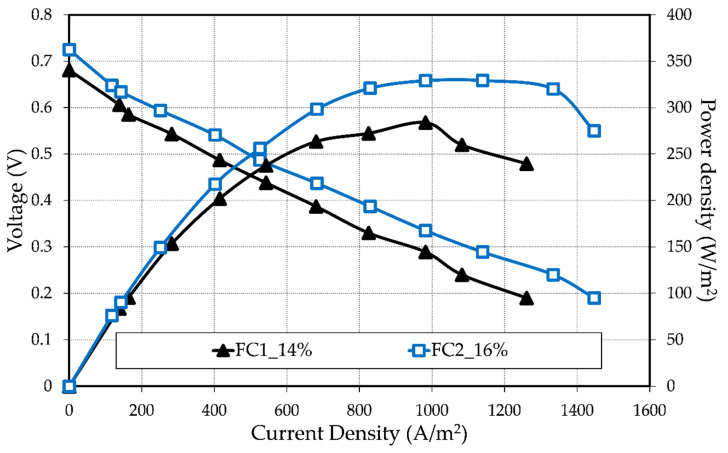
Polarization and power density of MEAs with different anode GDL porosities recorded after 5 h of operation at a constant load (T = 160 °C, ambient pressure, A = 4.78 cm^2^).

The performance differences were assumed to have an origin in the anode GDL due to the changes in the porosity since all other parts of the MEAs were manufactured identically. The porosity effect was evaluated through an electrochemical characterization of single tubular fuel cells operated with hydrogen and air as the fuel and oxidant, respectively. The measurements were made non-simultaneously; thence, some slight differences in operating conditions could have happened, especially regarding the gas channel temperature. The recorded polarization and calculated power density curves of the MEAs in Figure 5 correspond to its maximum performance, which was reached after 5 h of operation at a constant load of 0.06 A.

Although both MEAs had an acceptable OCV at the beginning of life (BOL), after 5 h of operation, it decreased considerably from 0.74 to 0.68 V and from 0.85 to 0.73 V for FC1_14% and FC2_16%, respectively. The OCV decrease relative to the theoretical OCV of 1.18 V (for steam) is known as a mixed loss and it is principally caused by a crossover of reactants. In an operated fuel cell, the OCV is affected not only by the reactant crossover, but also by the migration of some electrons through the membrane; the redistribution of H_3_PO_4_ during the first hours of operation; and the unwanted side reactions, such as carbon corrosion, metal corrosion, or/and passivation [13,38]. Therefore, the differences here presented in the OCV over time suggested a fuel cell degradation in both MEAs. Thus, a short-term stability test was conducted and is explained in the next section.

As can be seen in Figure 5, the performance of FC2_16% was higher than that of FC1_14%, indicating that an increase in porosity and pore size of the GDL led to a better overall performance of the fuel cell, as well as the importance of a smooth surface that is free of agglomerates. For example, at an operation current density of 209.21 A/m^2^ (= 0.1 A) the voltage was 0.57 V and 0.61 V for FC1_14% and FC2_16%, respectively. This enhancement in all current density regions is thought to be due to a better distribution of the hydrogen gas within the fuel cell. FC2_16% produced an increase in the current density of ≈200 A/m^2^ and a maximum power density of 329.25 W/m^2^.

**Figure 6:**
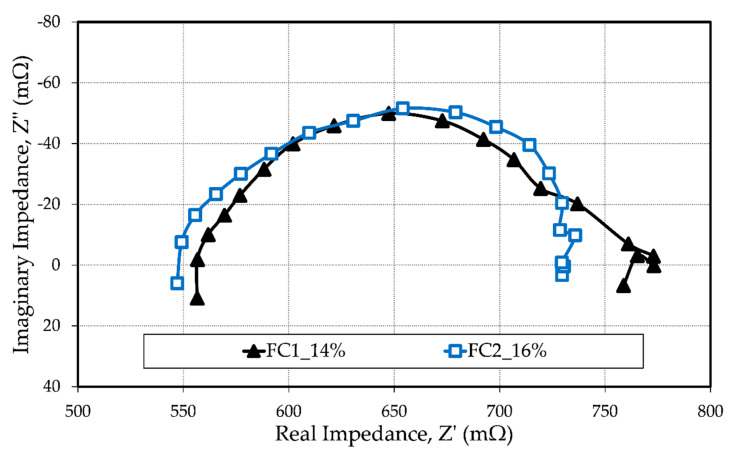
Nyquist plot for the EIS measurements at 0.06 A, T = 160 °C, ambient pressure, and A = 4.78 cm^2^ after 5 h of operation at a constant load.

The Nyquist plots in Figure 6 show the in situ electrochemical impedance measurements of the fabricated MEAs recorded at a constant load of 0.06 A. The intersection with the real axis indicates the membrane resistance, while the size of the arc corresponds to the electrochemical resistance.

Both MEAs showed a similar trend behavior with a slightly better fuel cell performance for FC2_16%. The high-frequency intercept with the x-axis shows that FC1_14% had a membrane resistance of 557 mΩ, while FC2_16% had a membrane resistance of 548 mΩ. The difference of 9 mΩ indicates that FC1_14% had a higher membrane resistance. Since both fuel cells were coated with the same membrane (same doping level) on the same day under the same environmental conditions, it is considered that the difference was caused by a loss of phosphoric acid from the membrane during operation. The size of the arcs also indicates that FC2_16% had a lower electrochemical resistance, i.e., a better electrochemical active layer, as a result of a better electrolyte distribution during operation and good interaction between the electrodes and the membrane. Table 1 summarizes the determined cell resistances, namely the membrane resistance (R_Ω_) and electrochemical resistance (R_ec_).

The EIS results show a very high fuel cell membrane resistance, even though both membranes were prepared with a high doping level. This can be attributed to the large thickness of the membrane used in this study, approx. 300 μm (uncompressed), which considerably surpasses the commercial sol-gel HT-PEMFCs [39,40,41]. The membrane production parameters were chosen in such a way that thick membranes were produced because it was found during preliminary tests that membranes thinner than 200 μm were prone to cause an electrical short-circuit.

### 3.3. Short-Term Stability Test

**Figure 7:**
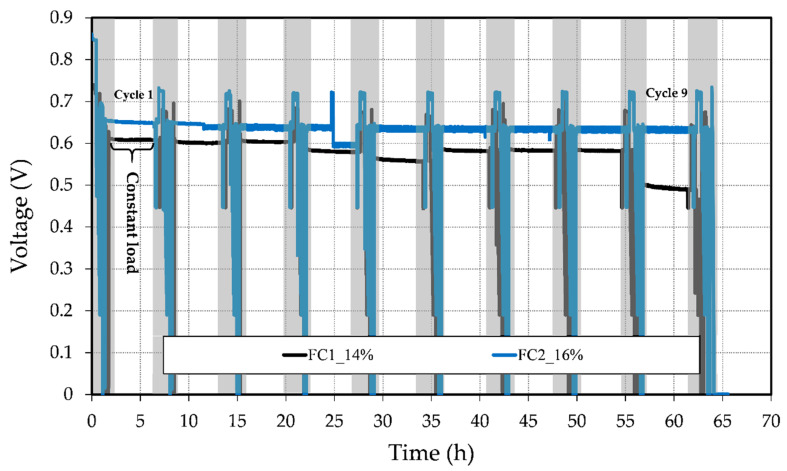
Stability test of the prepared MEAs with different anode GDL porosities.

To evaluate the OCV behavior and the possible degradation of the fuel cell during operation, a short-term stability test was conducted at a constant load of 0.06 A. This test was carried out after the fuel cell break-in over 45 h, interrupted every 5 h by in situ electrochemical measurements, which are indicated in Figure 7 with the gray boxes. The fuel cells were conditioned before each polarization curve and EIS measurement to prevent the test station from getting stuck. Figure 7 illustrates the change of voltage throughout the test period. It can be seen that for both fuel cells, the voltage degradation of the MEA and OCV decreased over time. Table 2 presents an overview of this behavior. The OCV drop corresponded to the difference between the OCV at the BOL and the end of the period test, while the voltage degradation rate was calculated using the voltage difference over the test period.

Although FC1_14% presented a slightly lower OCV drop from approximately 0.74 to 0.64 V than FC2_16% from 0.85 to 0.72 V, the latter showed significantly less voltage degradation throughout the test period with a rate of 0.511 mV/h, approximately 6 times lower than FC1_14%.

In Figure 7, it can be seen that FC1_14% exhibited a stronger descending trend; however, from cycle 5 to cycle 6, there was a variation and a better cell voltage was reached. This could have been caused by an internal reorganization of the electrolyte and generated water in the GDEs. During the fuel cell removal from the fuel cell reactor, an unexpected greenish liquid was found inside the anode channel. To classify the nature of the liquid, a pH measurement was carried out, indicating its high acidity. On this basis, it may be inferred that FC1_14% had an electrolyte leakage, which could be the principal reason for the strong voltage decline in the last cycle (cycle 9). The leakage may have been caused by the larger pore size on the catalyst layer and possible corrosion on the carbon support of the catalyst, i.e., a change in the catalyst layer hydrophobicity. Hence, the electrolyte could move easier through the layer, causing catalyst layer flooding and a reduction in the active reaction sites. Figure 7 also shows that FC2_16% had no abrupt voltage drops, except in constant load cycle 4. In this cycle, the test station had an internal error (the automatic load changed from 0.06 A to approx. 0.1 A). Despite the data fluctuation in FC2_16% increasing as the test progressed, the cell voltage remained relatively constant at ≈0.636 V. During the fuel cell operation, different degradation mechanisms took place, such as (i) the loss of active reaction sites due to particle agglomeration in the electrodes and carbon corrosion, (ii) membrane degradation, and (iii) phosphoric acid leaching [2,3,4,42]. The mechanism behind the decrease in the fabricated MEAs’ performance requires further investigation.

## 4. Conclusions

The application of a novel, 3D-printed, porous, thin-walled, 316L stainless steel tubular element as a gas diffusion layer with a tailored porosity in HT-PEMFC was demonstrated. The porosity influence on the fuel cell performance was evaluated using an electrochemical characterization of two corresponding multi-layer tubular HT-PEMFCs under usual operating conditions (160 °C, H_2_/air, ambient pressure).

In this study, the best performance was obtained with FC2_16%, showing a peak power density of 329.25 W/m^2^ and a voltage of 0.61 V at 0.1 A. This fuel cell also showed better stability throughout the short-term test period of 45 h. The strong decay in voltage for FC1_14% in the last 5 h of the test was due to an electrolyte leakage confirmed after the test period. Thus, it was concluded that an anode GDL with a porosity of 16% provided a better structure for fuel cell construction.

Although the MEAs were still below commercial planar HT-PEMFC performances (cf. References [3,43,44]), the fabricated fuel cells were comparable with other tubular designs found in the literature for both in HT-PEMFC [13] and LT-PEMFC [25,26,45] systems. Likewise, the engineered tubular electrodes introduced in this investigation represent a promising possibility for future research and development in fuel cell GDLs. To achieve greater porosities, and thus possibly better performances, fine-tuning of the PBF-LB process parameters is required. This could be done by exploring a new process window and other metallic materials. Additionally, research into a further reduction in membrane thickness is recommended to facilitate the mass-transport ability within the fuel cell.

## Figures and Tables

**Table 1:** Resistances for single tubular HT-PEMFCs.

Fuel Cell	Membrane Resistance, RΩ (mΩ)	Electrochemical Resistance, Rec (mΩ)
FC1_14%	557	216
FC2_16%	548	182

**Table 2:** OCV and voltage degradation over the test period.

Fuel Cell	OCV Drop (V)	Voltage Degradation Rate (mV/h)
FC1_14%	0.10	3.067
FC2_16%	0.13	0.511
